# Retraction: Protective effect of hsa‐miR‐570‐3p targeting CD274 on triple negative breast cancer by blocking PI3K/AKT/mTOR signaling pathway. Li‐Li Wang, Wei‐Wei Huang, Jing Huang, Rong‐Fang Huang, Na‐Ni Li, Yi Hong, Mu‐Lan Chen, Fan Wu, Jian Liu, Kaohsiung J Med Sci. 2020; 36(8): 581–591 (https://doi.org/10.1002/kjm2.12212)

**DOI:** 10.1002/kjm2.12790

**Published:** 2023-12-13

## Abstract

The above article, published online on 20 April 2020, and a corresponding corrigendum for Figure 7, published on 14 March 2022, in Wiley Online Library (wileyonlinelibrary.com), have been retracted by agreement between the authors, the journal Editor‐in‐Chief, Wan‐Long Chuang, and John Wiley and Sons Ltd.

The retraction has been agreed due to a high degree of duplication and similarity of the images presented in this manuscript. Original data for this manuscript is no longer available, Accordingly, the conclusions of this manuscript are not considered reliable.

The above article, published online on 20 April 2020, and a corresponding corrigendum for Figure 7, published on 14 March 2022, in Wiley Online Library (wileyonlinelibrary.com), have been retracted by agreement between the authors, the journal Editor‐in‐Chief, Wan‐Long Chuang, and John Wiley and Sons Ltd.

The retraction has been agreed due to a high degree of duplication and similarity of the images presented in this manuscript. Original data for this manuscript is no longer available, Accordingly, the conclusions of this manuscript are not considered reliable.

